# Optimization of the Composition of Silicone Enamel by the Taguchi Method Using Surfactants Obtained from Oil Refining Waste

**DOI:** 10.3390/polym13213619

**Published:** 2021-10-21

**Authors:** Vitaliy Tyukanko, Alexandr Demyanenko, Antonina Dyuryagina, Kirill Ostrovnoy, Marianna Lezhneva

**Affiliations:** Department of Chemistry and Chemical Technology, M. Kozybayev North Kazakhstan University, Petropavlovsk 150000, Kazakhstan; demianenkoav@mail.ru (A.D.); adyuryagina@inbox.ru (A.D.); kostrovnoy@mail.ru (K.O.); mlezhneva_@mail.ru (M.L.)

**Keywords:** dispersion, surfactants, silicones, polyorganosiloxanes, composition optimization, Taguchi method, enamels, organic coatings

## Abstract

The aim of this work is to optimize the composition of a two-component silicone enamel consisting of an aluminum pigment and a polyphenylsiloxane polymer to obtain the maximum dispersion of the pigment in the coating. The following products were used as surfactants: AS-1, PEPA, and Telaz. To assess the effect of surfactants on the dispersion of the pigment, computer-optical microscopy was used. The results of the studies showed that all the studied surfactants cause an improvement in the dispersion of the pigment. According to the degree of influence on the dispersion of the pigment, surfactants can be arranged in a row: PEPA > Telaz > AS-1. When the PEPA content in the enamel is 0.25 g/dm^3^, a decrease in the diameter of the pigment particles by 46% (from 26 to 14 microns) is recorded, with an increase in their specific amount by 2 times (from 258 to 550 pcs). Optimal enamel compositions allow a reduction in the corrosion rate by 11 times (from 0.6 to 0.053 mm/year) and improvement to the decorative properties of coatings (roughness, gloss, etc.). The effectiveness of the AS-1 product (obtained from oil refining waste) as a dispersant additive in silicone enamel has been proven.

## 1. Introduction

Silicone/polyorganosiloxane polymers are the basis (film-forming) for the production of heat-resistant paint and varnish materials, namely organo-soluble enamels and powder paints [1]. It is revealed that the duration of the protective life of silicone coatings at high temperatures is determined by a combination of a large number of factors, such as: the type of pigment, its dispersion in the coating [2], supramolecular structure (the presence and size of domain structures of polyorganosiloxanes in the coating), and the type of organosilicon polymers used [3,4]. The dispersion of pigments included in silicone enamels is the most important characteristic that determines the properties of coatings. For all investigated silicone composite materials (paints, plastics, and rubber), the following rule is applied: “the maximum of the protective and mechanical properties of the composites corresponds to the maximum dispersion of solid-phase pigments and fillers in the polyorganosiloxane matrix”. Introduction of aluminum nanoparticles, titanium dioxide, etc. to silicone material allows us to obtain more corrosion-resistant coatings [5]. In addition, silicone enamels require a very narrow technological range of coating thickness, due to the fact that with a small coating thickness, a long protective life of the painted steel surface is not provided. Additionally, when the recommended thickness is exceeded, the coating cracks (due to a catastrophic increase in internal stresses, with a temperature drop of several hundred degrees Celsius). Therefore, for silicone enamels, a very urgent issue is to increase their protective resource (when maintaining small thicknesses) what can be achieved by obtaining coatings with maximum pigment dispersion in a polyorganosiloxane matrix. The work of a number of authors shows an increase in the dispersion of pigments in coatings in the presence of surfactants [6].

In silicone paint and varnish materials, substances of polyester (PEG) and alkyd nature are used as surfactants [7,8]. However, the oxidation of PEG in seawater causes a significant reduction in the service life of the silicone coating. In [9], the effect of a new polyoxazoline (POS) additive on the properties of silicone coatings was investigated. It is shown that the introduction of POS makes it possible to obtain coatings with good anti-adhesive properties, despite the greater surface roughness and less compatibility with silicone polymer. It was shown in [10] that the introduction of 2-benzimidazolyl ethantiol into silicone enamel causes the formation of a highly effective antifouling coating.

The study [11] proves that the introduction of nanosilicon (SiO_2_) containing benzotriazole into polydimethylsiloxane makes it possible to obtain a self-healing coating. These coatings demonstrate increased protection of carbon steel from corrosion. An interesting study is presented in [12], showing that the introduction of an additive (adhesion enhancer) into a silicone composite can significantly increase its mechanical properties. Moreover, the high adhesive characteristics of the composite demonstrate good stability both when used for several hours and during many attachment/disconnection cycles. The study [13] showed that the introduction of the amino-containing product A-39 into the polyorganosiloxane compositions causes the formation of smaller supramolecular structures with a denser packaging. As a result, the coatings obtained are characterized by a longer protective resource. The introduction of surfactants into a composite based on polymethylsiloxane resin makes it possible to obtain protective coatings with greater pigment dispersion in the polymer matrix [14]. The coatings obtained in this case have a more ordered supramolecular structure and improved protective characteristics. The world scientific literature reports on the development of silicone composites and paints, using the following additives: aniline oligomer [15], 3-aminopropyltriethoxysilane [16], erucamide [17], melamine [18], polyamidoamine [19], amines of the amiture/ancamide series [20], and polyimides [21].

Therefore, the use of nitrogen-containing additives as surfactants to improve the dispersion of pigment in silicone enamels seems quite promising. Due to the deteriorating economic and environmental situation in the world, it becomes urgent to develop cheap additives obtained from chemical waste. As an additive in the study, an original industrial product obtained from oil refining waste—AS-1 was used, the synthesis of this product was carried out in the laboratory of the M. Kozybayev NKU [22]. In addition, to compare the assessment of the effect of AS-1 on pigment dispersion with effect of industrial additives produced on an industrial scale, the study used additional additives: “Telaz D Dispersant” and PEPA.

## 2. Materials and Methods

### 2.1. Materials

Polyphenylsiloxane varnish (hereinafter referred to as PPhS) was used as a polymer, the mass fraction of non-volatile substances is 17%; manufacturer: Spektr company, Russia. Toluene was used as a solvent (density 0.866 g/cm^3^ (at 20 °C); toluene content 99.80%; mass fraction of non-aromatic hydrocarbons 0.1%; manufacturer: Slavneft, Russia. The pigment used was aluminum pigment, PAP-2 grades; the content of active aluminum 97%; the content of iron 0.3%; the content of fat additives 2.5%, manufacturer: SUAL-PM, Russia. 

As nitrogen-containing surfactants, we used:

(1) The original industrial product obtained from oil refining waste—a mixture of primary and secondary amines AS-1 (molecular weight 250 a.m.u.; amine number (mg HCl/g) 30) was synthesized by our laboratory [22].
(2) The technical product of condensation of vegetable oils with diamines under the trademark “Telaz D Dispersant” (molecular weight 2121 a.m.u.; amine number (mg HCI/g) 32) manufacturer Avtokoninvest, Russia.
(3) A technical product, a mixture of high-molecular PEPA amines (molecular weight 4950 a.m.u.; amine number (mg HCI/g) 31), manufacturer JSC Uralkhimplast, Russia.

### 2.2. Computer-Optical Microscopy of the Dispersing Effect of Surfactants in Suspensions

To assess the effect of surfactants and solvent on the dispersion of pigment in the coating, it is necessary at first to evaluate their effect on the size of pigment particles in the suspensions “pigment-polymer-solvent“. This issue is quite complex and has various solutions: atomic force microscopy [23] of electrophoretic light scattering (ELS) [24], ultrasound research methods [25], and fluorescent and dark-field microscopy [26]. However, from a practical standpoint, the most effective method is the method of computer-optical microscopy [27]. Based on the above, we used a complex of computer-optical microscopy to assess the dispersion of the pigment.

To assess the effect of surfactants on the dispersion of the pigment in suspensions “aluminum pigment-toluene-PPhS”, the method of computer-optical microscopy was used. We used an installation consisting of a Pentium III personal computer, a CARL ZEISS 451422 microscope, and a USB camera. A special computer program, DSS (differential distribution spectrum), was developed for the installation of computer-optical microscopy. This program allowed us to process suspensions on the base of obtained microscope images. At the same time, the program determines the number of pigment particles (per unit area) and their geometric parameters (linear dimensions and area). The program calculates the content of various fractions of pigment particles in suspensions. Calibration of the installation on standardized objects showed that the measurement error does not exceed 5–6% (rel.). The duration of the analysis of one sample (from photographing to issuing the results) is 3–4 min.

### 2.3. Experimental Design

To plan the experiment (due to the large number of surfactant and solvent concentrations studied), the Taguchi method was used. The Taguchi method makes it possible to find the best combination of initial components for obtaining polymer composites with the necessary parameters [28,29]. In our case, it is necessary to obtain polyorganosiloxane enamel with maximum pigment dispersion in suspensions “aluminum pigment-toluene-PPhS”.

During the research, compositions were used in which were varied:

(1) The content of surfactants (hereinafter CS) from 0 to 0.5 g/dm^3^;
(2) The solvent content (hereinafter referred to as SC) from 10 to 50% (by weight of the composition).

Table 1 shows the factors under consideration and their corresponding levels [29].

#### 2.3.1. Method of Preparation of Suspensions for Computer-Optical Microscopy

(1) A total of 8.65 g of PPhS varnish were poured into a sealed reactor (volume (0.2 ± 0.01) dm^3^, filling factor 0.60).
(2) Toluene was added to the PPhS varnish. The SC ranged from 10 to 50%.
(3) Surfactant (CS), previously dissolved in toluene, was introduced in the specified quantities.
(4) An aluminum pigment of 0.35 g (3.5% of the mass of the mixture) was added.
(5) The reactor lid was hermetically closed, and the mixing device was turned on (an impeller agitator, speed of rotation 300min^−1^).
(6) After 30 min of mixing, the agitator was turned off and the lid was opened.
(7) Then, the suspension samples were placed on a slide using a pipette (a drop volume of 20 mL), fixed with a cover glass and held for 5 min under a static load of 10 g/cm^2^.
(8) After exposure for 5 min, the sample was subjected to computer-micro-optical scanning.

#### 2.3.2. The Method of Analysis of Suspensions

When performing the analyses, the multiplicity of magnification of the computer-optical system was set constant. The dispersion of the pigment was controlled by changes in the specific number of pigment particles on a fixed area (S_0_ = 0.04 mm^2^) of the processed image (SNP) and the average particle diameter of the pigment (APDP). The above characteristics were determined by the results of the analysis of five parallel samples.

#### 2.3.3. Planning an Experiment Using the Taguchi Method

In this study, the L9 matrix was used, and the signal-to-noise ratio (S/N) was used as a characteristic of the selection quality. In the Taguchi method, three different functions are used to find the signal-to-noise ratio (S/N): “the-smaller-the-better” (SB), “the-nominal-the-best” (NB), or “the-larger-the-better” (LB). To minimize APDP, we used the SB function (Equation (1)), and to maximize SNP, we used the LB function (Equation (2)) [29].

(1) $$S/N_{S}=-10\log_{10}\left(\frac{1}{n}\overset{n}{\underset{i=1}{\sum }}y_{i}^{2}\right)$$

(2) $$S/N_{L}=-10\log_{10}\left(\frac{1}{n}\overset{n}{\underset{i=1}{\sum }}\frac{1}{y_{i}^{2}}\right)$$

where *n* is the number of test runs and *y* is the measured dispersion values of APDP or SNP.

The *S/N_S_* ratios for APDP were calculated according to Equation (1), as shown in Table 2.

The *S/N_L_* ratios for SNP were calculated according to Equation (2), as shown in Table 3.

### 2.4. Methodology for Checking the Quality of Coatings

The corrosion rate was determined in the Caspian Sea water according to ISO 11845:2020(en) standard on steel plates 65 cm × 25 cm × 0.1 cm (length × width × thickness). The coating was applied to the plates by the filling method and kept for 8 h at a temperature of 350 ± 5 °C. The test time was 96 h. The corrosion rate was determined by gravimetric method (weighing the plates before and after exposure in the water of the Caspian Sea). Each sample was weighed at least 5 times. The results show the average values.

The appearance of the coatings was evaluated on steel plates measuring 15 cm × 7 cm (length × width) in natural (sunlight) light. The coating was applied to the plates by the filling method and kept for 8 h at a temperature of 350 ± 5 °C. All samples were examined at least 5 times.

## 3. Results

### 3.1. Effect of Surfactants on Pigment Dispersion

**Figure 1:**
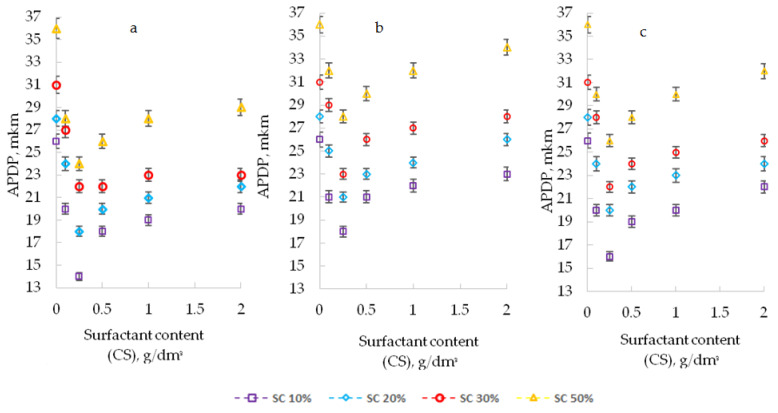
Influence of the type and content of surfactants (CS) on ADPD at different SC (10–50%); (**a**), PEPA; (**b**), AS-1; and (**c**), Telaz.

**Figure 2:**
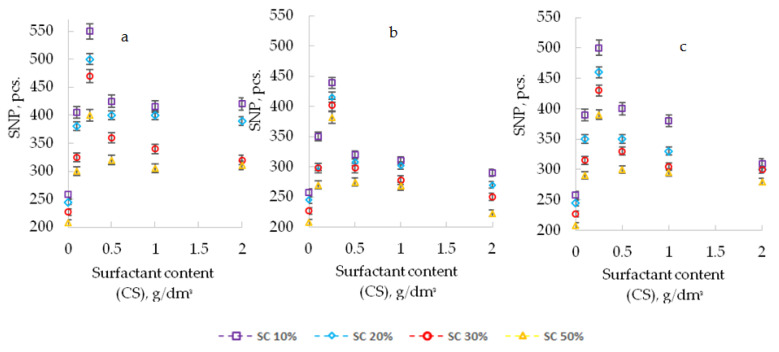
Influence of the type and content of surfactants (CS) on SNP at different SC (10–50%); (**a**), PEPA; (**b**), AS-1; and (**c**), Telaz.

**Figure 3:**
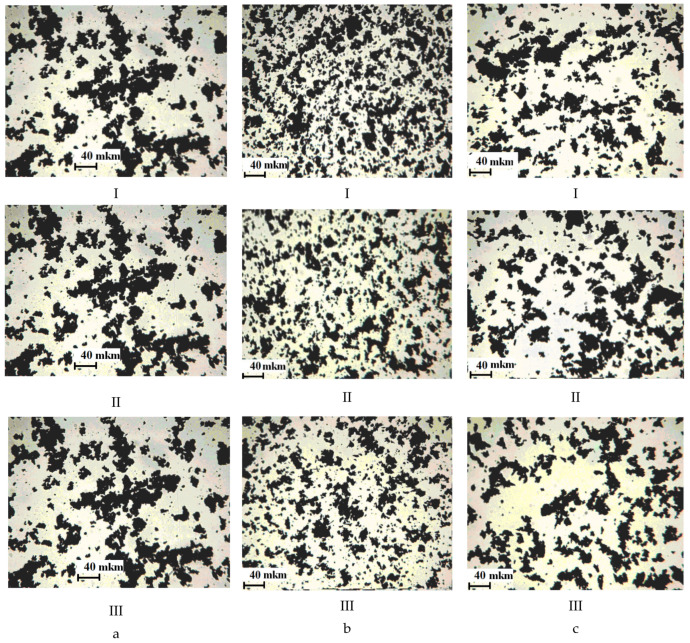
Micrographs of PPhS suspensions with different CS: I, PEPA; II, Telaz; and III, AS-1 for CS, g/dm^3^: (**a**), 0 g/dm^3^; (**b**), 0.25 g/dm^3^; and (**c**), 0.5 g/dm^3^.

When surfactants were introduced into the composition, an improvement in the dispersion of the pigment was confirmed by computer-optical microscopy, which is expressed in a decrease in the diameter of the pigment particles (APDP) and an increase in their number (SNP) (Figure 1, Figure 2 and Figure 3).

Figure 3 shows photos of suspensions obtained by computer-optical microscopy, with different content of additives.

**Figure 4:**
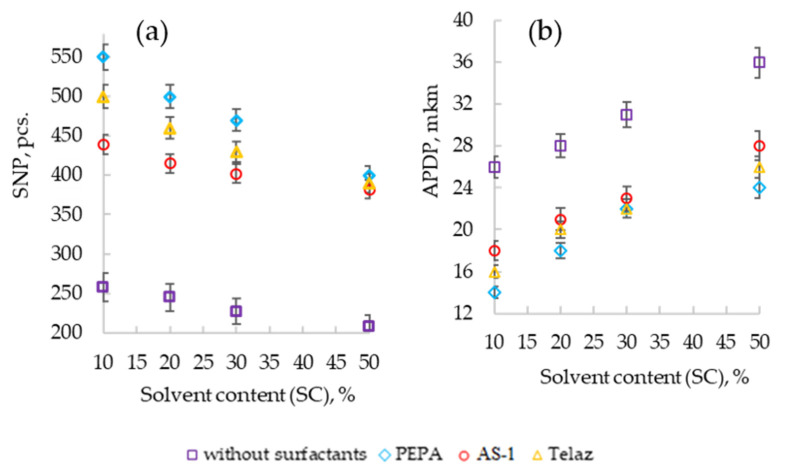
Effect of the surfactant type on APDP (**a**) and SNP (**b**) at CS = 0.25 g/dm^3^.

The effect of the solvent content on the dispersion of the pigment is shown in Figure 4.

With an increase in the solvent content in the composition, computer-optical microscopy revealed a deterioration in the dispersion of the pigment, expressed in an increase in the diameter of the pigment particles (APDP) Figure 4a and a decrease in their number (SNP) Figure 4b.

### 3.2. Optimization of the Composition of Polyphenylsiloxane Enamel by the Taguchi Method

The average values of the signal-to-noise ratio for each parameter level for APDP are shown in Table 4, for SNP are shown in Table 5.

The values of rank and delta determined the most significant parameter for optimizing the dispersion of the pigment. From Table 4, it was found that the solvent content (SC) is the most significant parameter affecting APDP, followed by CS (surfactant content). However, for SNP, CS is the most significant optimization parameter.

To assess the contribution of each factor to the parameters of pigment dispersion, an analysis of variance (ANOVA) was provided, the calculation results are presented in Table 6 and Table 7.

ANOVA analysis when evaluating the dispersibility of the pigment showed that for APDP (Table 6), the most significant optimization parameter is the solvent content (SC) contribution of 61.27%, and for SNP (Table 7), the determining parameter is CS (surfactant content) contribution of 91.15%.

To derive a multifactorial statistical mathematical model of the effect of surfactants and solvent content on APDP and SNP, the equation proposed by M. M. Protodiakonov (3) was used:

(3) $$Y_{0}=\frac{\prod_{i=1}^{n}Y_{i}}{Y_{M}^{n-1}}$$

where $Y_{0}$ is the generalized equation; $Y_{i}$ is the particular function; $\prod_{i=1}^{n}Y_{i}$ is the product of all particular functions; *n* is the number of particular functions equal to the number of input parameters; and $Y_{M}^{n-1}$ is the total average of all the considered values of the generalized function to a degree one less than the number of particular functions.

The reliability of the obtained mathematical model is determined by calculating the coefficient of nonlinear multiple correlation [18]:

(4) $$R=\sqrt{1-\frac{\left(n-1\right)\cdot \sum_{i=1}^{n}{\left(y_{i}-{\hat{y}}_{i}\right)}^{2}}{\left(n-p-1\right)\cdot \sum_{i=1}^{n}{\left(y_{i}-\bar{y}\right)}^{2}}},$$

where n is the number of experiments; p is the number of input (independent) parameters; i is the serial number of the experiment; $y_{i}$ is the actual value of the output parameter in the i experiment; ${\hat{y}}_{i}$ is the calculated value of the output parameter, calculated using a multi-factor mathematical model, for the conditions (values of input parameters) of the *i* experiment; and $\bar{y}$ is the average value of the actual value of the output parameter for all *n* experiments (the general average).

After approximating the dependencies, using Microsoft Excel, the equation for APDP is obtained:

(5) $$APDP=\frac{\left(a\cdot CS^{2}+b\cdot CS+c\right)\cdot \left(d\cdot SC+e\right)}{APDP_{M}}$$

where *APDP* is the average diameter of pigment particles in microns; *SC* is the solvent content as a percentage; and *CS* is the surfactant concentration in solution, g/dm^3^.

Table 8 shows the coefficients included in Equation (5) for various surfactants.

After approximating the dependencies, the equation is obtained for the SNP:

(6) $$SNP=\frac{\left(a\cdot CS^{2}+b\cdot CS+c\right)\cdot \left(d\cdot SC+e\right)}{SNP_{M}}$$

where *SNP* is the specific number of pigment particles on a fixed area (S_0_ = 0.04 mm^2^), pcs. *SC* is the solvent content as a percentage; and *CS* is the surfactant concentration in solution, g/dm^3^.

Table 9 shows the coefficients included in Equation (6) for various surfactants.

The reliability of the obtained mathematical models was estimated by calculating the coefficients of nonlinear multiple correlation (4). The minimum coefficient of nonlinear multiple correlation among the proposed mathematical models is 0.989.

### 3.3. The Effect of Surfactants on the Quality of Coatings

**Figure 5:**
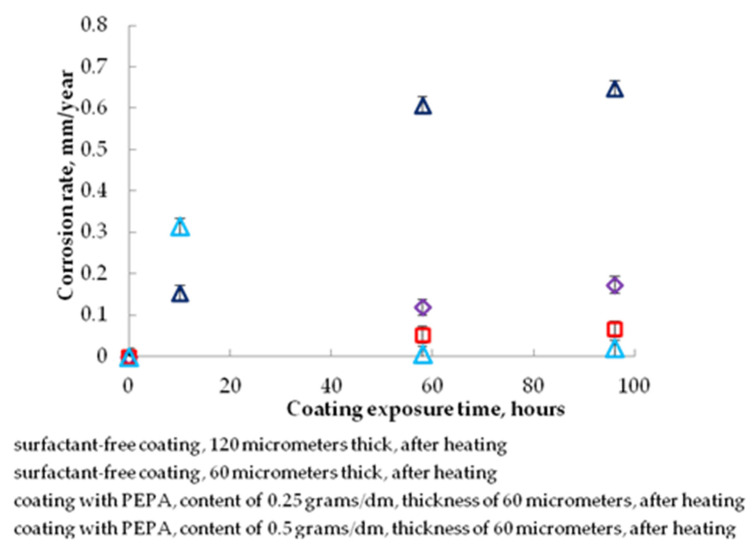
The effect of the surfactant content (CS) on the corrosion rate of the coating obtained from the recommended enamel.

Due to the fact that the maximum dispersion of the pigment is fixed for the PEPA (at 0.25 g/dm^3^ contents), the quality of the coatings is checked only with this surfactant. The effect of surfactants on the corrosion rate under the coating is shown in Figure 5.

**Figure 6:**
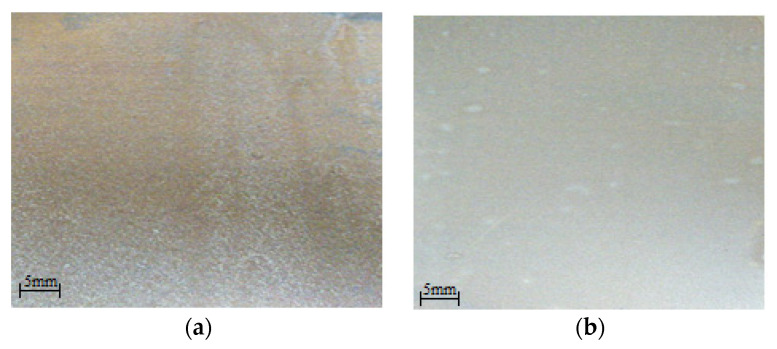
The effect of the content of PEPA on the appearance of the coating; (**a**) without surfactants; and (**b**) with a PEPA of 0.25 g/dm^3^.

The introduction of PEPA into the enamel causes a decrease in the corrosion rate, thereby increasing the protective life of the coating. The effect of the PEPA on the appearance of the coating is shown in Figure 6. Visually, the roughness of the coating has significantly decreased.

## 4. Discussion

The improvement of pigment dispersion is recorded for all the studied additives. The maximum dispersion of the pigment was recorded at CS 0.25 g/dm^3^ for all the studied surfactants. For a PEPA at CS 0.25 g/dm^3^, the APDP decrease was 46% (from 26 to 14 microns), and the SNP increased by 2 times (from 258 to 550 pcs). In similar conditions for AS-1, the decrease in APDP was 31%, and the SNP increased by 1.7 times. For Telaz, the decrease in APDP was 38.5%, and the SNP increased by 1.9 times. Therefore, the three studied additives according to the quantitative indicators of the dispersing effect (APDP and SNP) can be arranged in a row (followed in descending order of activity): PEPA > Telaz > AS-1. The most effective surfactant dispersant for polyphenylsiloxane enamel is PEPA. 

As the solvent content in the composition (SC) increases, computer-optical microscopy revealed a decrease in the dispersing effect of surfactants, expressed in an increase in the diameter of pigment particles (APDP) and a decrease in their number (SNP) (Figure 1, Figure 2 and Figure 3). However, the presence of additives in the enamel allows us to minimize the harmful effect of the solvent. With an increase in the solvent content (SC) from 10 to 50% in suspensions (without surfactants), APDP increases from 26 to 36 microns (by 38.5%), and SNP decreased from 258 to 208 pcs. (by 20%). With the introduction of additives in the composition, it is possible to stabilize the dispersing effect of surfactants. Thus, PEPA at a concentration of 0.25 g/dm^3^ with an increase in SC from 10 to 50% stabilizes the APDP value at a level of up to 22–24 microns (without surfactants up to 36 microns), and SNP decreases to 400 pcs. (without surfactants up to 208 pcs.). Therefore, even in highly dilute solutions of polyphenylsiloxanes, it is possible to stabilize the dispersion of the pigment due to the optimal selection of the type and content of the additive.

**Figure 7:**
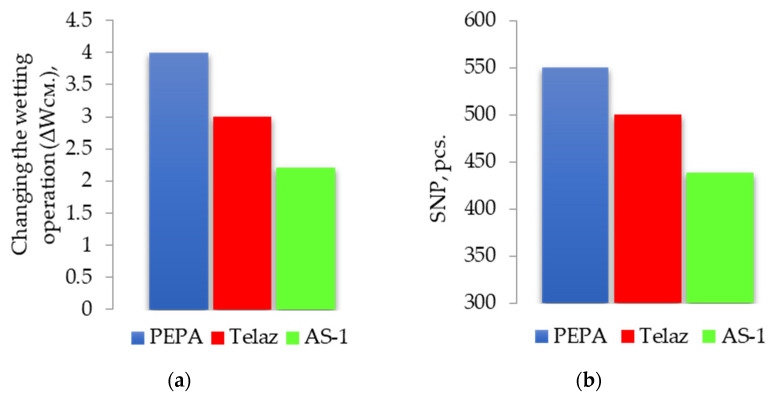
The effect of additives on the change in the wetting operation (ΔW_cm_) with respect to (**a**) the aluminum substrate (SC 10%) and (**b**) the dispersion of the pigment (SNP, pcs.) at CS 0.25 g/dm^3^ and a temperature of 25 °C [30].

To explain the dispersing effect of additives, we consider the effect of surfactants on the surface tension of solutions of polyphenylsiloxanes and wetting of aluminum pigment, described by us in [30]. We have previously proven the surface activity of the studied additives in a polyphenylsiloxane composition at the liquid–gas interface (“polyphenylsiloxane solution/air”) [30]. The minimum surface energy for solutions of polyphenylsiloxane and all surfactants was observed at CS 0.25 g/dm^3^. The effect of surfactants on the change in wetting performance in industrial varnish (SC10%) is closely correlated with the results of our study of the dispersibility of pigment presented on Figure 7.

According to Figure 7, the maximum increase in ΔW_cm_ is recorded for PEPA on the pigment/aluminum substrate; at the same time, it is characterized by the greatest dispersing activity. Thus, the given content of additives in the compositions provides an improvement in the wetting of the pigment with solutions of polyphenylsiloxane. Based on all of the above, it can be argued that the studied additives exhibit surface activity at the interface of the “liquid/gas” phases (reducing the surface energy of solutions of polyphenylsiloxanes) and “liquid/solid” (improving the wetting of the pigment), as a result, there is an improvement in the dispersion of the pigment in the enamel. The obtained results can be explained by the possible adsorption of the studied additives on the active/adsorption centers of the pigment. 

**Figure 8:**
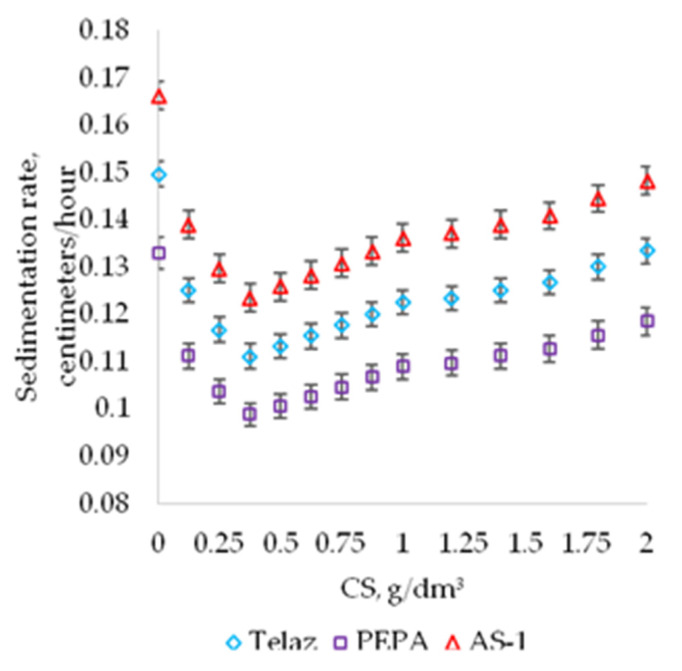
The effect of CS on the sedimentation rate of suspensions “aluminum pigment—PPhS—toluene” (centimeter/hour) at SC 10%.

The dispersing activity of the studied additives, revealed by computer-optical microscopy, is confirmed by the previously shown decrease in the sedimentation rate of suspensions, presented on Figure 8.

The three studied surfactants according to the degree of influence on the sedimentation rate of suspensions can be arranged in a row (followed in descending order of stability): PEPA > Telaz > AS-1, which is completely consistent with the results of the study of the dispersing activity of surfactants by computer-optical microscopy.

**Figure 9:**
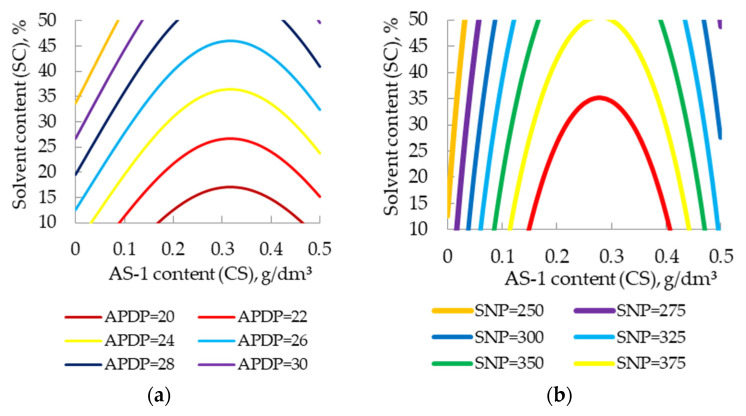
Two-factor nomograms: (**a**) SNP = *f* (SC, CS); (**b**) APDP = *f* (SC, CS).

Using the Taguchi method, and on the basis of Equations (5) and (6), the optimal surfactant and solvent contents in the enamel were determined, ensuring maximum dispersion of the pigment. For the obtained additive AS-1, nomograms for finding APDP and SNP from known surfactant and solvent contents are presented in Figure 9.

The introduction of the studied additives into polyphenylsilaxane enamel causes an 11-fold decrease in the corrosion rate (from 0.6 to 0.053 mm/year). A similar effect of reducing the corrosion rate can be achieved without the use of surfactants by simply increasing the coating thickness (from 60 to 120 microns) from 0.6 to 0.12 mm/year. However, in this case, the coating will have a significantly low protective resource due to its cracking. The increase in the anticorrosive resistance of the coating, with an increase in the content of PEPA in the coating from 0.25 to 0.5 g/dm^3^, can be explained by the inhibitory activity of the introduced additives, primarily amines, which can be adsorbed on the active corrosion centers of the steel substrate and shield them. The effect of the PEPA on the dispersion of the pigment can be seen from Figure 6; as a result, the decorative properties (roughness, gloss, etc.) should improve in the coating. 

A logical continuation of this study is to study the influence of the considered additives on the protective and decorative properties of coatings. It would be especially interesting to check the effect of surfactants on the heat resistance of coatings (by thermogravimetry, in the temperature range from 100 to 450 °C).

## 5. Conclusions

(1) The investigated surfactants: PEPA, Telaz, and AS-1 improve pigment dispersion. The most effective surfactant dispersant of the studied additives is PEPA. There is a close correlation between dispersing the effect of the studied surfactants and the previously described change in the wetting operation.
(2) The Taguchi method optimized the composition of silicone enamel, which allowed us to maximize the dispersion of the pigment. ANOVA analysis showed that the dispersibility of the pigment is significantly influenced by both studied factors. For APDP, the most significant optimization parameter is the solvent content (SC) contribution of 61.27%, and for SNP, the CS (surfactant content) contribution of 91.15%.
(3) The optimal enamel composition (CS = 0.25 g/dm^3^ and SC = 10%) reduces the corrosion rate by 11 times (from 0.6 to 0.053 mm/year). In addition, this enamel composition provides smoother surfaces.
(4) The proposed product AS-1 can be successfully used as an additive-dispersant for polyphenylsiloxane enamels. The dispersing activity of AS-1, in the studied enamel, is close to industrial additives, PEPA and Telaz dispersants. The original product, AS-1, is synthesized from oil refining waste, which is one of the ways to reduce the burden on the environment (less emissions of substances will pollute nature).

## Figures and Tables

**Table 1:** Parameters and levels for the Taguchi method.

Factors	Level 1	Level 2	Level 3
CS (g/dm^3^)	0	0.25	0.5
SC (wt.%)	10	20	50

**Table 2:** *S/N_S_* ratios of SB for APDP.

Trial	CS (g/dm^3^)	SC (wt.%)	S/N Ratio for APDP,(dB)	APDP, Micrometer
1	0	10	−28.299	26
2	0	20	−28.943	28
3	0	50	−31.126	36
4	0.25	10	−25.105	18
5	0.25	20	−26.444	21
6	0.25	50	−28.943	28
7	0.5	10	−26.444	21
8	0.5	20	−27.235	23
9	0.5	50	−29.542	30

**Table 3:** *S/N_L_* ratios of LB for SNP.

Trial	CS (g/dm^3^)	SC (wt.%)	S/N Ratio for SNP, (dB)	SNP, pcs.
1	0	10	48.232	258
2	0	20	47.783	245
3	0	50	46.361	208
4	0.25	10	52.849	439
5	0.25	20	52.361	415
6	0.25	50	51.641	382
7	0.5	10	50.103	320
8	0.5	20	49.771	308
9	0.5	50	48.787	275

**Table 4:** S/N ratios of APDP by parameter level (SB).

Level	S/N Ratio	
	CS (g/dm^3^)	SC (wt.%)
1	−29.456	−26.616
2	−26.831	−27.541
3	−27.740	−29.870
Delta	2.625	3.254
Rank	2	1

**Table 5:** S/N ratios of SNP by parameter level (LB).

Level	S/N Ratio	
	CS (g/dm^3^)	SC (wt.%)
1	47.459	50.395
2	52.284	49.972
3	49.554	48.930
Delta	4.825	1.465
Rank	1	2

**Table 6:** ANOVA for S/N ratios (for APDP).

Factor	Degree of Freedom	Sum of Square	Contribution of Factor (%)
CS (g/dm^3^)	2	10.662	38.73
SC (wt.%)	2	16.868	61.27
Total	4	27.530	100

**Table 7:** ANOVA for S/N ratios (for SNP).

Factor	Degree of Freedom	Sum of Square	Contribution of Factor (%)
CS (g/dm^3^)	2	35.12	91.15
SC (wt.%)	2	3.41	8.85
Total	4	38.53	100

**Table 8:** The value of the coefficients in Equation (5) for various surfactants.

№	Surfactant	*a*	*b*	*c*	*d*	*e*	*APDP_M_*
1	РЕРА	112	-73.333	30	0.2308	17.179	23.333
2	Тelaz	93.333	-60.667	30	0.2372	18.231	24.556
3	AS-1	80	-50.667	30	0.2423	19.205	25.667

**Table 9:** The value of the coefficients in Equation (6) for various surfactants.

№	Surfactant	*a*	*b*	*c*	*d*	*e*	*SNP_M_*
1	PEPA	−2784	1681.3	237	−2.5115	434.31	367.333
2	Telaz	−2504	1478	237	−2.0692	400.85	345.667
3	AS-1	−2288	1272	237	−1.2385	349.69	316.667

## Data Availability

The datasets generated during and/or analysed during the current study are available from the corresponding author on reasonable request.
